# Longitudinal assessment of magnetization transfer ratio, brain volume, and cognitive functions in diffuse axonal injury

**DOI:** 10.1002/brb3.2490

**Published:** 2022-02-01

**Authors:** Fabiola Bezerra de Carvalho Macruz, Fabrício Stewan Feltrin, Ana Zaninotto, Vinícius Monteiro de Paula Guirado, Maria Concepcion Garcia Otaduy, Miriam Harumi Tsunemi, Mariana Penteado Nucci, Carolina Rimkus, Celi Santos Andrade, Claudia da Costa Leite

**Affiliations:** ^1^ Department of Radiology and Oncology Hospital das Clínicas, Faculdade de Medicina da USP São Paulo Brazil; ^2^ Neuropsychology Division Department of Neurology Hospital das Clínicas, Faculdade de Medicina da USP São Paulo Brazil; ^3^ Neurosurgery Division Department of Neurology Hospital das Clínicas, Faculdade de Medicina da USP São Paulo Brazil; ^4^ Department of Biostatistics Universidade Estadual Paulista, Botucatu São Paulo Brazil

**Keywords:** axonal/myelinic damage, brain atrophy, diffuse axonal injury, magnetization transfer imaging, traumatic brain injury

## Abstract

**Background:**

Diffuse axonal injury (DAI) is a frequent mechanism of traumatic brain injury (TBI) that triggers a sequence of parenchymal changes that progresses from focal axonal shear injuries up to inflammatory response and delayed axonal disconnection.

**Objective:**

The main purpose of this study is to evaluate changes in the axonal/myelinic content and the brain volume up to 12 months after TBI and to correlate these changes with neuropsychological results.

**Methods:**

Patients with DAI (*n* = 25) were scanned at three time points after trauma (2, 6, and 12 months), and the total brain volume (TBV), gray matter volume, and white matter volume (WMV) were calculated in each time point. The magnetization transfer ratio (MTR) for the total brain (TB MTR), gray matter (GM MTR), and white matter (WM MTR) was also quantified. In addition, Hopkins verbal learning test (HVLT), Trail Making Test (TMT), and Rey–Osterrieth Complex Figure test were performed at 6 and 12 months after the trauma.

**Results:**

There was a significant reduction in the mean TBV, WMV, TB MTR, GM MTR, and WM MTR between time points 1 and 3 (*p* < .05). There was also a significant difference in HVLT‐immediate, TMT‐A, and TMT‐B scores between time points 2 and 3. The MTR decline correlated more with the cognitive dysfunction than the volume reduction.

**Conclusion:**

A progressive axonal/myelinic rarefaction and volume loss were characterized, especially in the white matter (WM) up to 1 year after the trauma. Despite that, specific neuropsychological tests revealed that patients’ episodic verbal memory, attention, and executive function improved during the study. The current findings may be valuable in developing long‐term TBI rehabilitation management programs.

## Graphical abstract

This manuscript evaluates longitudinal changes in the axonal/myelinic content and the brain volume of 25 patients after diffuse axonal injury. After trauma, the patients were scanned at three time points (2, 6, and 12 months). In addition, neuropsychological evaluation, more specifically the Hopkins verbal learning test, the Trail Making Test, and the Rey–Osterrieth Complex Figure test, were also performed in these patients within 6 and 12 months after the trauma.

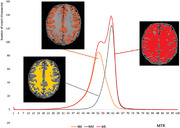

## 1 INTRODUCTION

Traumatic brain injury (TBI) is a public health and socio‐economic problem worldwide (Roozenbeek et al., 2013). In the United States, around 2.8 million new cases are diagnosed each year, with approximately 57,000 deaths (Frieden et al., 2015). In addition, 5.3 million people are estimated to live with a TBI‐related disability in the United States, accounting for around 2% of the population (Akin et al., 2020; Frieden, 2015).

Diffuse axonal injury (DAI), a type of lesion characterized by microscopic axonal damage scattered throughout the encephalic parenchyma secondary to acceleration‐deceleration forces, is one of the most common pathological mechanisms of TBI (Adams et al., 1989). DAI has been almost universally demonstrated in fatal TBI and is estimated to be the mechanism most likely responsible for many cognitive deficits resulting from moderate to severe TBI (Skandsen et al., 2010).

Although magnetic resonance imaging (MRI) is more sensitive for the detection of axonal shear injuries than computed tomography (CT) and the currently preferred modality for assessing parenchymal involvement in DAI (Liu et al., 2014), it significantly underestimates the brain alterations. Furthermore, up to now, there is no well‐established MRI sequence that can accurately evidence the severity of DAI or precisely monitor the progression of the post‐traumatic brain damage (Cole et al., 2018; de La Plata et al., 2007).

The need for alternative MRI techniques that reflect the complex underlying pathophysiological processes in DAI and allow reliable follow‐up of the brain damage has grown after experimental evidence of delayed cerebral changes after TBI (Irimia et al., 2012; Mamere et al., 2009). Examples of these techniques are the magnetization transfer imaging (MTI) and the brain volumetry.

The magnetization transfer ratio (MTR), a quantitative measurement obtained from the MTI, has been described as a reliable marker for monitoring myelinic and axonal content in the gray matter (GM) and white matter (WM) after TBI (Bagley et al., 2000; Gareau et al., 2020). Clinical studies have described a reduction in the MTR in the normal‐appearing white matter (WM) up to years after TBI. However, few of these studies include moderate and severe TBI, and in these, the MTR quantification is limited to small regions of interest (ROI) (Kumar et al., 2003; Mamere et al., 2009). In addition, they include a heterogeneous group of subjects with cortical contusions, hematomas, and ischemic injuries under the more general term “TBI”. Up to our knowledge, there are only two studies in patients exclusively with DAI (Bagley et al., 2000; Sinson et al., 2001), and none of these include neuropsychological assessment.

The number of studies describing volume change of various neuroanatomic structures in the chronic phase after TBI is higher, and progressive encephalic volume reduction is well‐established (Bigler, 2001; Cole, 2018; Tate, 2000). Nonetheless, the association of DAI with other intracranial lesions is also observed in most studies, and few are restricted to subjects exclusively with DAI (Ding et al., 2008; Moen et al., 2012; Warner et al., 2010).

Finally, DAI damages crucial networks between the cortex and the deep white matter structure. This injury leads to cognitive deficits, especially non‐specific memory impairment that can involve the encoding and retrieval of verbal and visual information equally (Levin, 1990; Palacios et al., 2013). Cognitive symptoms are more severe just after an injury and may only be present for a short time (Li et al., 2006; Zaninotto et al., 2017). Given the importance of verbal learning and memory in education and academic achievement, a significant number of studies have looked into the effects of TBI on verbal memory, but few have explored visuospatial memory after brain injury (Shum et al., 2000; Zaninotto et al., 2017).

## 2 MATERIALS AND METHODS

### 2.1 Study population

This was a 1‐year prospective longitudinal study for which approval was obtained from the Institutional Review Board. In addition, written informed consent was obtained from all participants or their representatives. Out of the 225 consecutive patients with TBI admitted to the Emergency Department who were initially considered for this study, 186 were excluded for not meeting the epidemiological, clinical, or radiological eligibility criteria.

The inclusion criteria were the following: (1) age between 18 and 55 years, (2) a TBI history dating with no longer than three months before the inclusion of the patient in the study, (3) an admission Glasgow Coma Scale (GCS) score of 3−12, (4) clinical and computed tomography (CT) diagnosis of DAI, and (5) a Marshall score of I, II, or III (Marshall et al., 1992) based on the CT images. The exclusion criteria were the following: (1) presence of contusion(s) larger than 10 mm or that may impair automated brain volume segmentation, (2) a midline shift greater than 0.5 cm, (3) extra‐axial fluid collection(s) with a compressive effect on the brain structures, or (4) any contraindications to brain MRI.

The remaining 39 patients who followed the abovementioned criteria were submitted to an initial neuropsychiatric (NP) evaluation and a brain MRI. Out of these, 13 abandoned the study in the follow‐up period, and one patient died. Thus, the final sample included 25 patients that were prospectively evaluated at three time points: around 2 months after the trauma (1), 6 months after the trauma (2), and 12 months after the trauma (3). Due to the comprehension difficulties, mental confusion, and agitation typically seen in the subacute stage post‐trauma, the patients were submitted to neuropsychological assessment only at time points 2 and 3.

### 2.2 Image acquisition

All data were acquired on a 3T MRI scanner (Phillips Achieva, Best, The Netherlands) with an eight‐channel head coil. Besides the sequences detailed below, a routine protocol that included fluid‐attenuated inversion recovery (FLAIR), T2‐weighted images, and diffusion‐weighted imaging (DWI) was also acquired.

The volumetric T1‐weighted Fast Field Echo (3DT1‐FFE) sequence was obtained in the sagittal plane with the following parameters: inversion time (IT) = 700 ms, TR/TE = 6.2 ms/2.7 ms; flip angle = 8°; acquisition matrix = 240 × 240; field of view (FOV) = 240 × 240 × 180 mm; voxel resolution = 1 mm^3^ (isotropic); slice thickness = 1.0 mm (180 slices). Susceptibility weighted image was acquired in the axial plane with a Principles of Echo Shifting with a Train of Observations (PRESTO) 3D‐T1FFE sequence; TR/TE = 22/29 ms; flip angle = 10°; FOV = 220 × 182 mm; matrix = 224 × 224; voxel size = 0.98 × 0.98 × 1.0 mm; slice thickness = 1.0 mm (130 slices).

Additionally, a set of two consecutive axial T1‐weighted 3D gradient‐echo pulse images was obtained (TR/TE = 3.6/7.3 ms, flip angle = 8°, FOV = 256 mm, acquisition matrix = 240 × 240; reconstruction matrix = 480 × 480; acquisition voxel = 1.0 × 1.0 × 3.0 mm; reconstruction voxel = 0.5 × 0.5 × 3.0 mm, 48 slices of 3.0 mm each, and acquisition time = 4 min), one without (*M*_0_) and one with an off‐resonance saturation pulse (*M*_1_) to assess magnetization transfer images (Fjær et al., 2013).

### 2.3 Image processing

**FIGURE 1:**
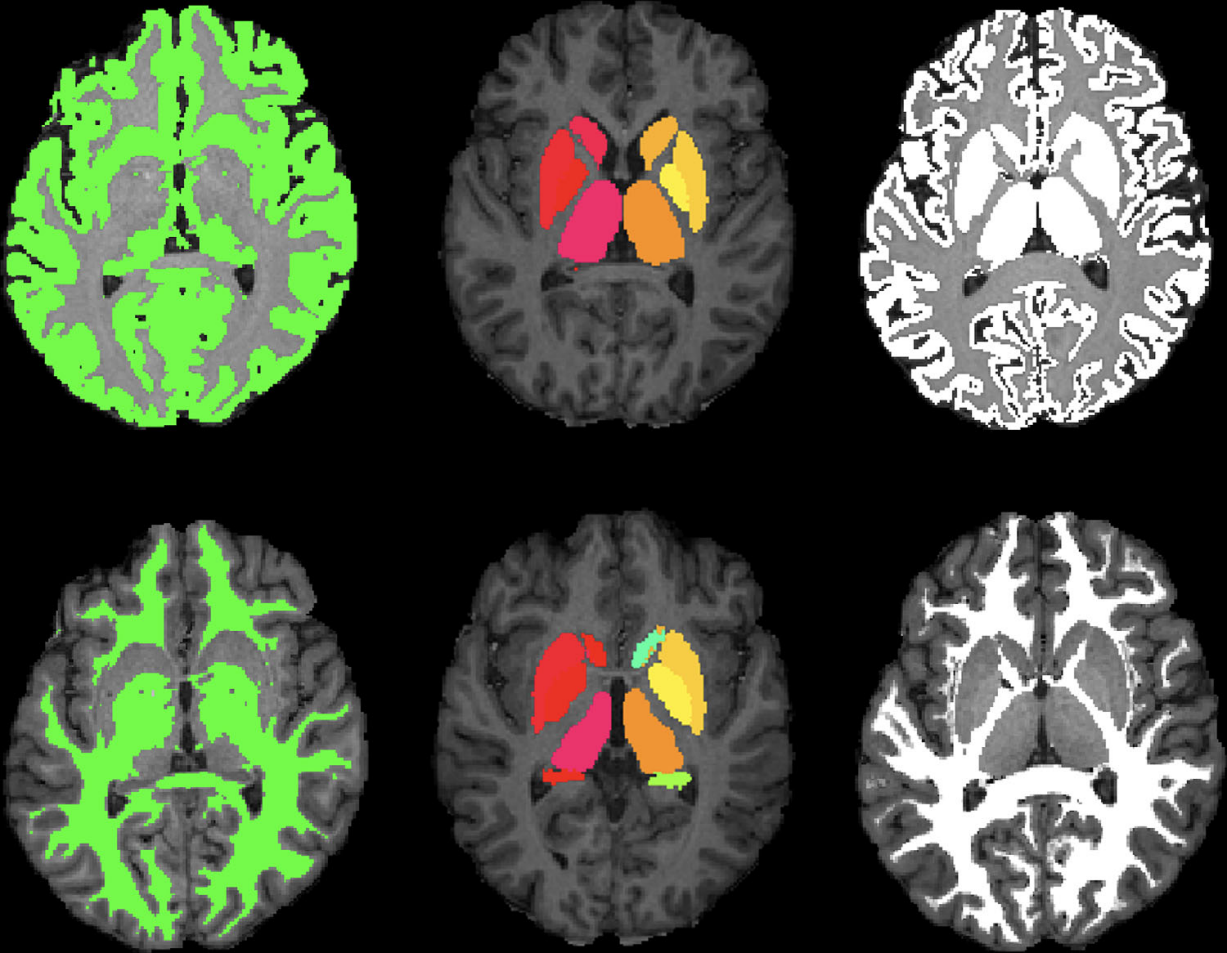
Automatic segmentation of the gray matter (GM) (top left) and the white matter (WM) (bottom left) using FAST from FSL. By applying a detailed subcortical GM mask generated by FIRST (middle) to the original masks, we attained a much more robust segmentation of the GM (top right) and the WM (bottom right)

The MTR was quantified using the FMRIB Software Library (FSL ‐ Analysis Group, FMRIB, Oxford, UK; available at http://www.fmrib.ox.ac.uk/fsl/) version 5.0. For that, segmentation of the GM and WM was performed in the anatomical 3D T_1_ using the FMRIB Automated Segmentation Tool (FAST). Then, the automatic segmentation generated by FAST had to be corrected using a more robust mask of the subcortical GM (amygdala, hippocampus, caudate, putamen, pallidum, and thalamus) obtained with the FMRIB's Integrated Registration and Segmentation Tool (FIRST) (Patenaude et al., 2011). From this process, we attained accurate T1‐based volumetric images corresponding to the GM, WM, and total brain (TB) from each MRI study (Figure 1).

**FIGURE 2:**
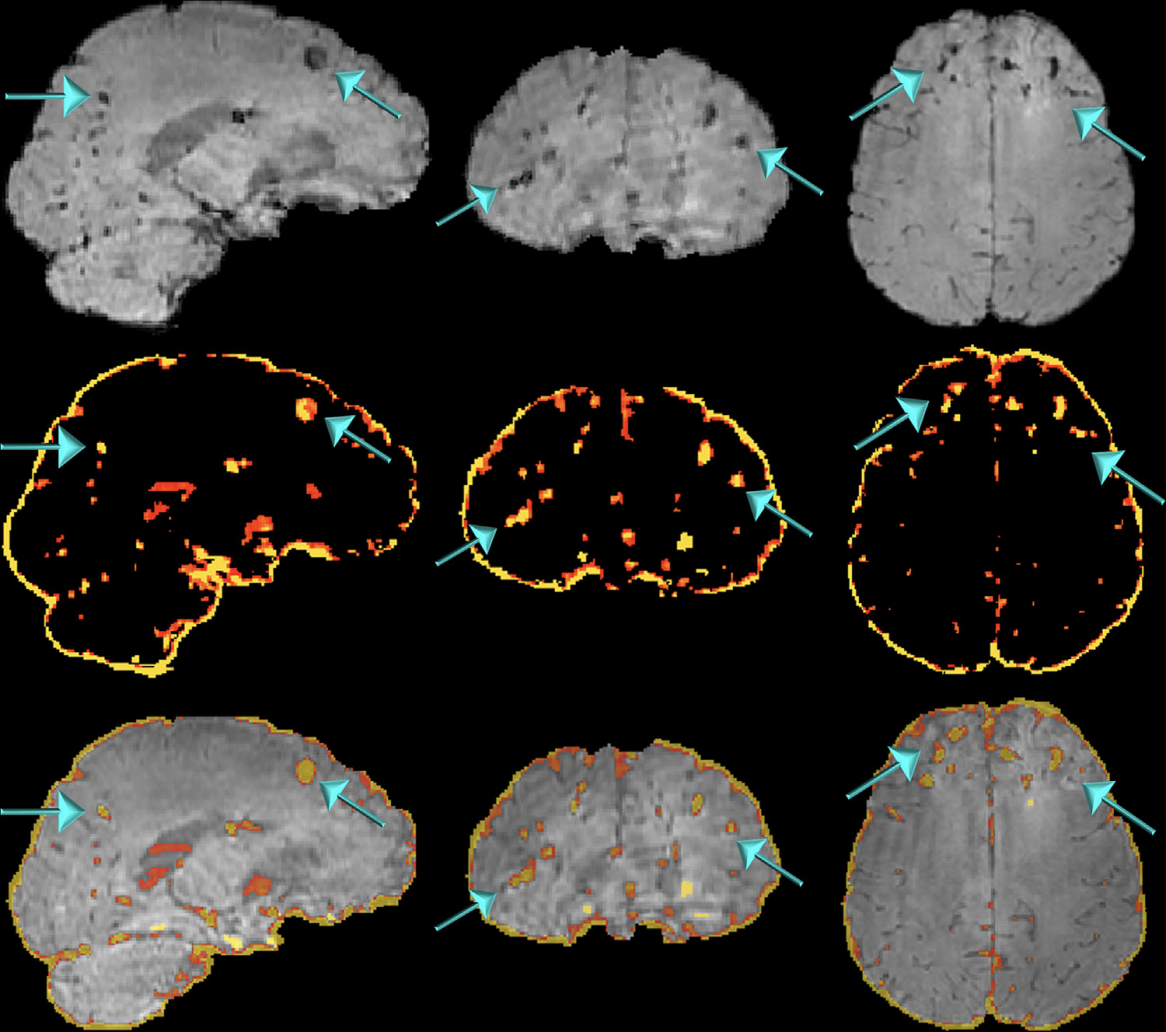
PRESTO images of a patient evidencing the hemorrhagic lesions (top). Automatic segmentation of the hemorrhagic lesions was obtained by applying a threshold to the PRESTO images based on the intensity of the voxels (middle). When the generated mask containing the hemorrhagic foci is superimposed over the original images (bottom), the technique's robustness is better evidenced

**FIGURE 3:**
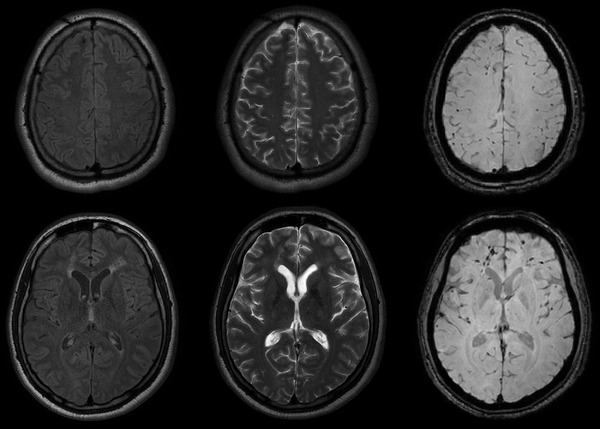
Axial MRI images of a patient evidencing small hemorrhagic lesions characteristics of diffuse axonal injury in the FLAIR (left), T2‐weighted (middle), and PRESTO images (right)

Second, we used the PRESTO images for the exclusion of the microhemorrhages from the MTR map. In the MTI processing, blood content is a major inconvenience since it corrupts the results by decreasing the MTR, and ideally, should be excluded from the MTR quantification. To accomplish that, we segmented the neural tissue from the PRESTO images into four different classes according to voxel intensity using FAST to obtain a mask containing only voxels with very low signals (Figure 2). Voxels with very low signal in the gradient echo images correspond to paramagnetic deoxyhemoglobin, methemoglobin, and hemosiderin (Chavhan et al., 2009). The microbleeds characteristic of DAI are almost imperceptible in other sequences (Figure 3). Using the affine transformation matrix resultant from the registration between the PRESTO and the T1 images, we could subtract the microbleeds mask from the GM, WM, and TB masks.

**FIGURE 4:**
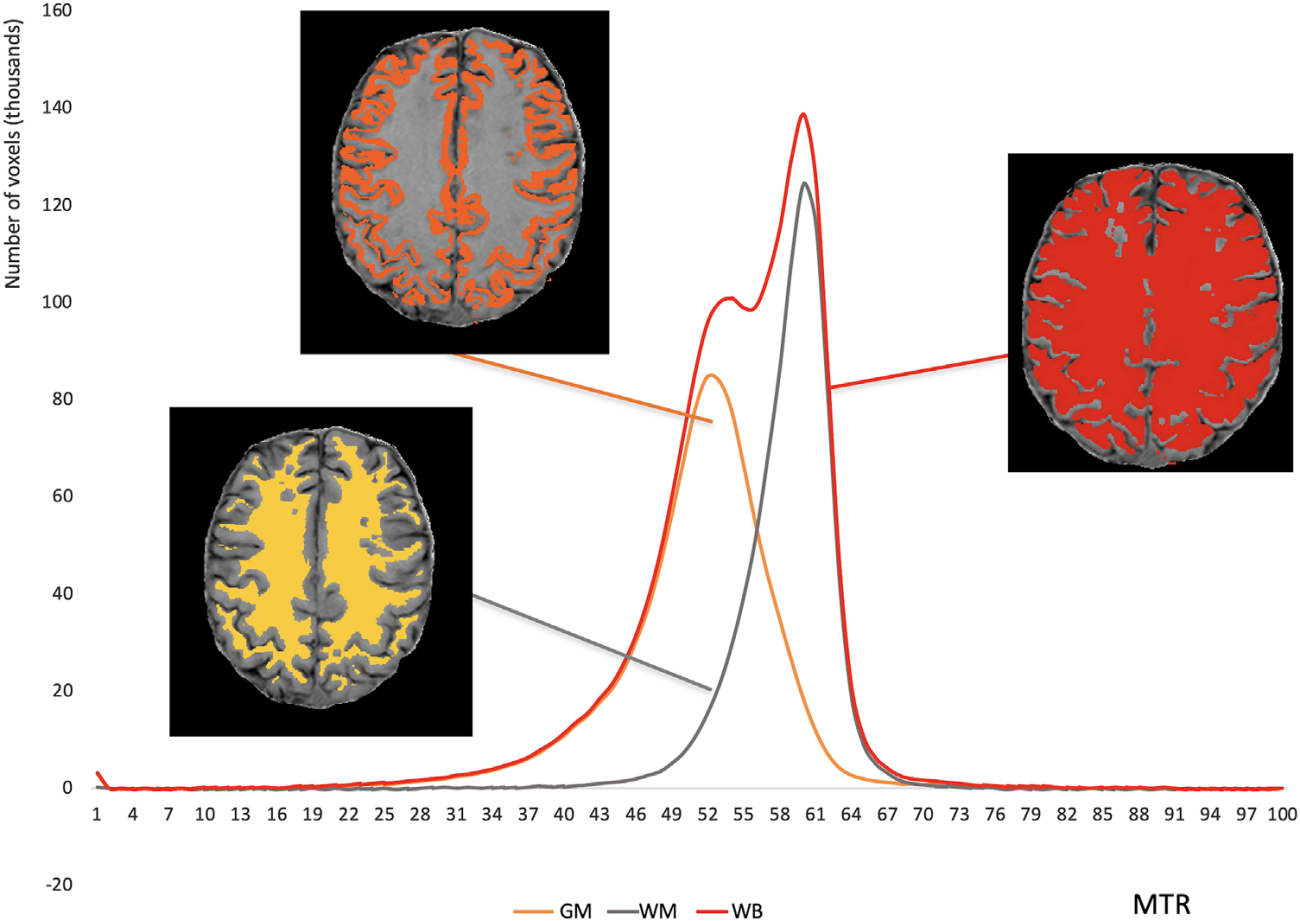
Example of the MTR histograms obtained from a time point of a patient. The histograms derived from the total brain (red), gray matter (orange), and white matter (yellow) are superimposed. The mean magnetization transfer ratio (MTR) for the total brain (TB MTR), the gray matter (GM MTR), and white matter (WM MTR) were further attained from these histograms

Third, the acquired MTI was separated into two data sets, obtained before (*M*_0_) and after a saturation pulse (*M*_1_). Then, the MTR map was computed applying the formula: [(*M*_0_ − *M*_1_) / *M*_0_] × 100. Because the MTR map has low contrast and poor definition, instead of segmenting the brain tissue directly in the MTR map, we carried the segmentation in the *M*_1_ image and the resulting mask applied to the MTR map. The final step of the MTI processing consisted of registering the brain extracted *M*_1_ image to the T1 image and using the inverse transformation matrix to register the GM, WM, and WB masks to the MTR map. For this study, we generated MTR histograms with 100 bins of size 1, and their mean was attained for the TB (TB MTR), the GM (GM MTR), and the WM (WM MTR) (Figure 4).

Volume quantification based on the T1‐weighted images was performed using the longitudinal pipeline of the FreeSurfer software (http://freesurfer.nmr.mgh.harvard.edu) version 7.1.1. FreeSurfer is a suite of tools for analyzing and visualizing structural and functional neuroimaging data that allows automated parcellation of the cortical grey and white matter and subcortical volumes (Fischl, 2012). Its longitudinal processing enables a temporally unbiased evaluation of an arbitrary number of time points by treating all inputs the same and generating a within‐subject template via iterative alignment of all input images to a median image, using a symmetric robust registration method (Reuter et al., 2012). Due to the long‐time processing of the pipeline's first step (*recon‐all* command), GNU Parallel was used, which allows multiple jobs to be performed simultaneously, one per core of the used computer, in our case, six at a time (Tange, 2011). The mean time for *recon‐all* part of the processing, which alone includes 31 steps, was ∼6 h per image; using GNU Parallel 4, processing results for six T1‐weighted images were obtained in this same time. Following FreeSurfer recommended best practices; we performed an individual inspection of the output files. For this study, the total brain volume (TBV), white matter volume (WMV), and gray matter volume (GMV) were measured.

### 2.4 Neuropsychological assessment

#### 2.4.1 Hopkins verbal learning test (HVLT)

This test evaluates the episodic verbal memory and consists of a 12‐item word list which is read to subjects on three successive learning trials. Free recall scores are recorded for each learning trial (HVLT‐I). Approximately 20−25 min later, a delayed recall trial (HVLT‐D) and a recognition trial (HVLT‐R) are completed. The delayed recall requires free recall of any words remembered. The recognition trial comprises 24 words, including the 12 target words and 12 confounding words, six semantically related, and six semantically unrelated (Benedict et al., 1998). The results are expressed as the total raw score.

#### 2.4.2 Trail Making Test (TMT)

Assesses attention and executive function. The test consists of two parts (A and B), one with numbers and the other with numbers and letters. In Part A, the task is to connect the numbers in ascending order (1‐2‐3‐4…). In Part B, subjects are required to alternately connect numbers and letters in ascending order for the numbers and sequential order for the alphabet letters (1‐A‐2‐B‐3‐C…). The time to complete each part is used when analyzing performance (Spreen & Strauss, 1998). The results are expressed as the total raw score.

#### 2.4.3 Rey–Osterrieth Complex Figure (ROCF) test

Evaluates the visuoconstructional ability and long‐term visual memory (nonverbal memory). It consists of a timed (but not time‐limited) trial in which subjects copy a complex two‐dimensional geometric figure shown on a stimulus trial (ROCF copy). After three minutes, individuals are required to draw the figure again from memory (ROCF recall). Individuals are not forewarned that they will be asked to recall the figure they have copied. This test provides a numerical scoring system based on the presence or absence of structural elements in the individual reproduction of the figure. The results are expressed as the total raw score.

#### 2.4.4 Intelligence quotient (IQ)

Was calculated by combining the vocabulary and matrix reasoning tests present in the WAIS‐III (Reid‐Arndt et al., 2011). The vocabulary test consists of the presentation of words, and the patient is asked to define them. In the matrix reasoning test, a matrix of abstract pictures with one picture missing is presented, and the patient has to choose an option that better suits the missing image.

### 2.5 Statistical analysis

Statistical tests were performed using the software RStudio version 1.4.1103 (2009‐2021). The Shapiro‐Wilk test assessed the distribution of the data, and a *p*‐value > .05 was considered normal. The Friedmann test investigated differences in the mean MTR and the volume between the three time points. Differences in the neuropsychological tests between the two time points were assessed using the paired t‐test (if the results were normally distributed) and the nonparametric Wilcoxon signed‐ranked test (if the results were not normally distributed). The neuropsychological assessment at each time point was reported using the raw score that represents the number of words (HVLT‐I, HVLT‐D, HVLT‐R), seconds (TMT‐A and TMT‐B), or attributes (ROCF copy and ROCF recall). Pearson test (if the results were normally distributed) or Spearman test (if the results were not normally distributed) were used to calculate correlation coefficients. Results were considered significant if *p*‐value < .05.

Multiple linear regression models assessed the relationship between the independent variables (age at trauma and the time from trauma to first scan) and the dependent variables (volume reduction and MTR reduction between time‐points), and a *p*‐value < .05 was considered significant. For this analysis, we used the atrophy rate or the MTR decline rate corrected by a 1‐year interval (%/year) to compensate for the different scan intervals from the first to the last scan in different patients. Multiple linear regression models also assessed the relationship between the independent variables (age, years of education, IQ, annual volume reduction, and annual MTR decline rates) and the neuropsychological scores, with a *p*‐value < .05 considered significant.

## 3 RESULTS

### 3.1 Demographics

A total of 25 outpatients (mean age = 28.36 years, SD = 9.17 years) with a history of moderate or severe DAI according to the GCS score at admission (<13) completed the 3 MRI scans. The majority of these patients were male (*n* = 22), suffered from motorcycle accidents (*n* = 14), presented with severe head trauma (*n* = 16) and a Marshall score of II on the initial CT (*n* = 21). The mean time interval between trauma and hospital admission was 39 min (SD = 16.50). Table 1 provides basic demographic data and clinical characteristics.

**TABLE 1:** Description of the dataset

**Characteristics**		***n* (SD)**
Patients	Gender	—
	‐ Male	22
	‐ Female	3
	Age (years)	28.36 (9.17)
	Education (years)	10.2 (2.82)
Trauma	Severity of trauma according to the GCS score on admission	—
	‐ Moderate (GCS 9–12)	9
	‐ Severe (GCS 3–8)	16
	Mechanism of trauma	—
	‐ Motorcycle accident	14
	‐ Car accident	6
	‐ Running‐over	4
	‐ Physical aggression	1
	Interval between trauma and hospital admission (minutes)	39 (16.50)
Initial CT	Marshall grading	—
	‐ Category I	4
	‐ Category II	21
MRI scan	Trauma to time point 1 interval (days)	53.52 (22.84)
	Trauma to time point 2 interval (days)	194.48 (30.03)
	Trauma to time point 3 interval (days)	373.32 (24.97)

*Abbreviations*: GCS, Glasgow coma scale; MRI, magnetic resonance imaging; *n*, number; SD, standard deviation.

One patient did not perform the neuropsychological assessment on time point 2, and three did not perform the NP assessment on time point 3. These four patients were excluded from the longitudinal NP evaluation and the correlation analysis between imaging findings and neuropsychological performance but were included in the imaging evaluation.

### 3.2 Volumetric and MTR results

**FIGURE 5:**
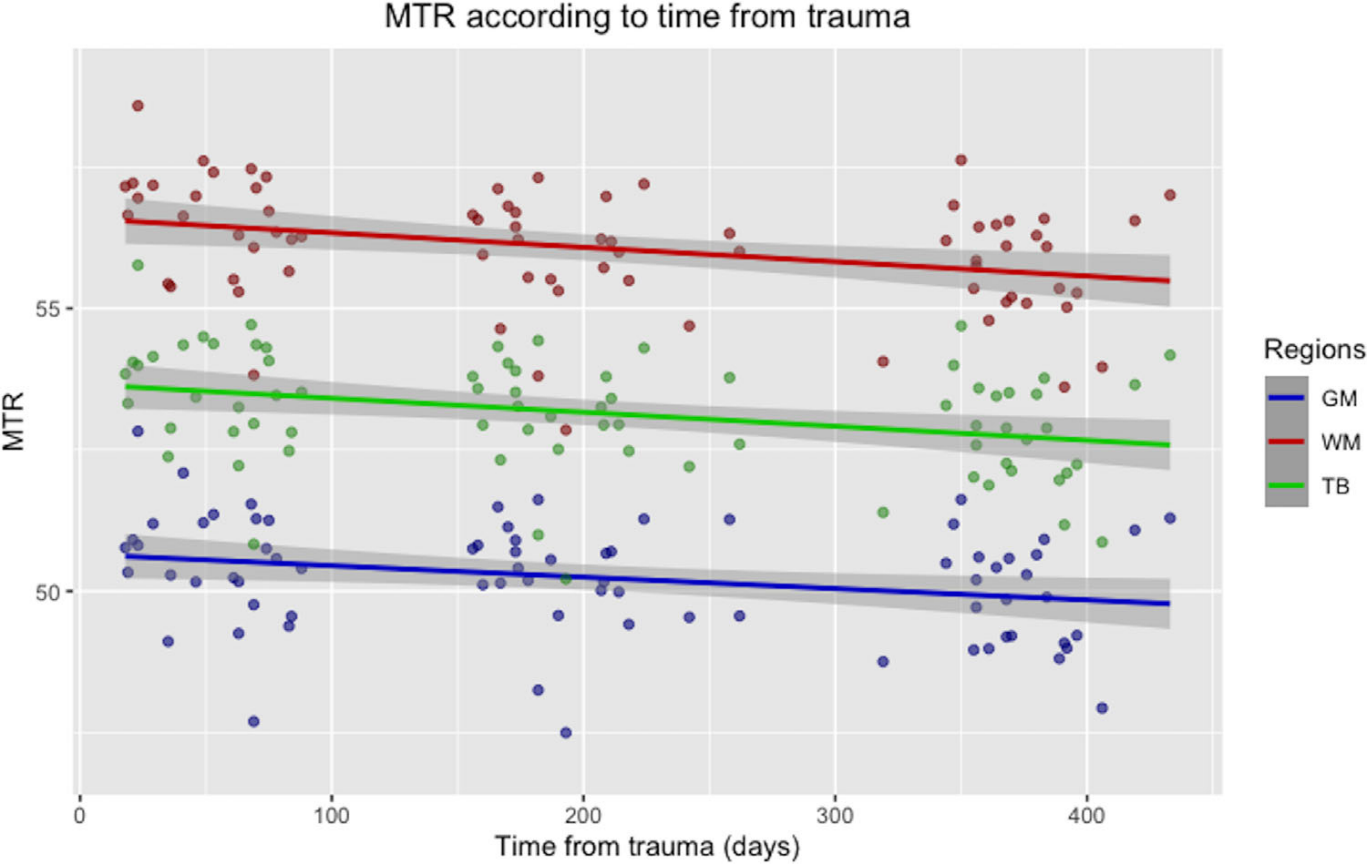
Scatter plot with regression line showing the magnetization transfer ratio (MTR) change over time for the gray matter (GM), white matter (WM) and total brain (TB). The *x*‐axis represents the time from trauma in days, and the *y*‐axis represents the mean MTR

**FIGURE 6:**
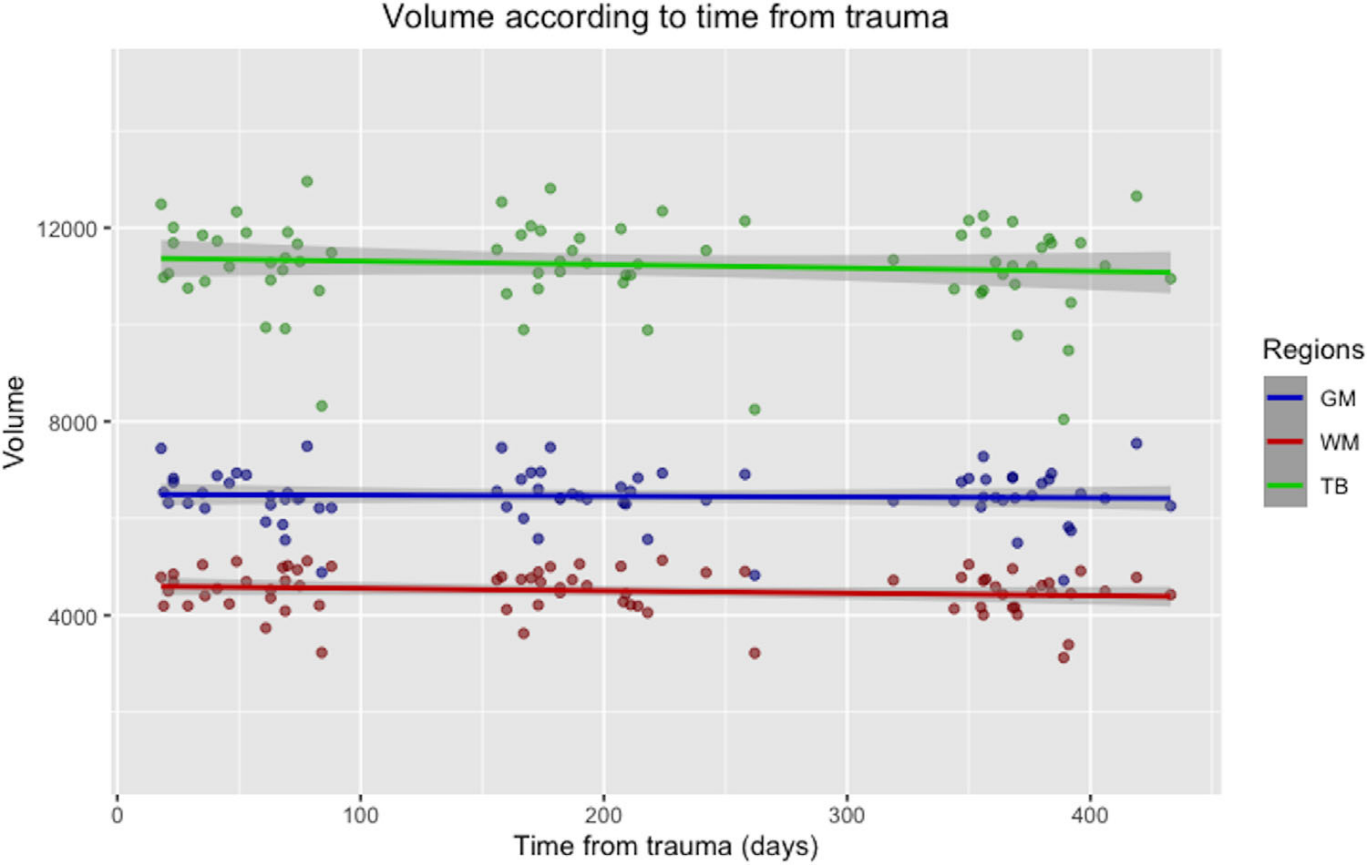
Scatter plot with regression line showing the volume change over time for the gray matter (GM), white matter (WM) and total brain (TB). The *x*‐axis represents the time from trauma in days, and the *y*‐axis represents the volume

Table 2 summarizes the volumetric and MTR data as mean, standard deviation, maximum, and minimum values for each time point. Figures 5 and 6 show the MTR change and the volume change, respectively, according to the time after trauma. Between time points 1 and 3, there was a significant decline in the mean TBV, WMV, TB MTR, GM MTR, and WMV MTR. Between time points 1 and 2, there was a significant reduction in the mean WM MTR. Between time points 2 and 3, there was a significant decrease in the mean TBV, WMV, and GM MTR. The mean GMV did not differ significantly across time points.

**TABLE 2:** Brain segmentation and Friedmann test results

**Variable**	***n***	**Time point**	**Mean**	**SD**	**Minimum**	**Maximum**	**Comparison**	***p*‐value**
TBV	25	1	1,127,180.640	93,629.350	832,052.000	1,295,790.000	**1** **vs. 2**	.906
TBV	25	2	1,129,439.800	96,138.586	824,814.000	1,281,122.000	**1** **vs. 3**	**.02**
TBV	25	3	1,114,438.160	98,536.506	804,310.000	1,265,143.000	**2** **vs. 3**	**0**
GMV	25	1	644,228.985	54,521.290	488,161.945	749,025.717	**1** **vs. 2**	.157
GMV	25	2	648,446.985	57,089.768	482,593.927	746,706.723	**1** **vs. 3**	.989
GMV	25	3	645,742.425	57,369.164	471,839.006	754,855.898	**2** **vs. 3**	.117
WMV	25	1	455,165.720	45,831.197	322,742.000	512,086.000	**1** **vs. 2**	.989
WMV	25	2	453,332.680	46,160.541	321,856.000	513,709.000	**1** **vs. 3**	**0**
WMV	25	3	441,752.800	45,514.703	31,2577.000	504,951.000	**2** **vs. 3**	**0**
TB MTR	25	1	53.552	1.011	50.833	55.764	**1** **vs. 2**	.206
TB MTR	25	2	53.097	0.988	50.217	54.430	**1** **vs. 3**	**0**
TB MTR	25	3	52.782	0.977	50.868	54.690	**2** **vs. 3**	.086
GM MTR	25	1	50.517	1.055	47.696	52.825	**1** **vs. 2**	.957
GM MTR	25	2	50.269	0.954	47.497	51.619	**1** **vs. 3**	**.005**
GM MTR	25	3	49.918	0.967	47.933	51.622	**2** **vs. 3**	**.013**
WM MTR	25	1	56.532	0.987	53.821	58.583	**1** **vs. 2**	**.013**
WM MTR	25	2	55.929	1.063	52.849	57.311	**1** **vs. 3**	**0**
WM MTR	25	3	55.725	0.994	53.608	57.623	**2** **vs. 3**	.495

*Abbreviations*: GMV, gray matter volume; TBV, total brain volume.

The linear regression analysis showed that none of the independent variables (age at trauma, year of education, and time from trauma to the first scan) had a significant influence on the annualized TB MTR, GM MTR, or WM MTR (*p* > .05). They also didn't have a significant influence on the annualized TBV, GMV, WMV atrophy (*p* > .05).

### 3.3 Neuropsychological assessment

Table 3 summarizes the neuropsychological findings. In time point 3, patients completed the TMT‐A and TMT‐B significantly faster than in time point 2. They could also remember considerably more words in the HVLT‐I in time point 3 than in time point 2, but not in the HVLT‐D or HVTL‐R. There were no significant differences in IQ, the ROCF copy test, or the ROCF recall test results between time points.

**TABLE 3:** Raw scores of the neuropsychological tests and *t*‐test (Normality = 0) or Wilcoxon test (Normality = 1) results

**Test**	**Time point 1 Mean (SD)**	**n1**	**Time point 2 Mean (SD)**	**n2**	**Normality**	***p*‐value**
IQ	85.25 (10.77)	24	88.00 (10.31)	22	0	.495
HVLT‐I	18.45 (4.69)	24	22.04 (5.56)	22	0	**.011**
HVLT‐D	5.16 (2.63)	24	5.95 (2.91)	22	0	.334
HVLT‐R	9.29 (2.33)	24	9.81 (2.21)	22	1	.313
TMT‐A	53.58 (35.39)	24	44.77 (35.68)	22	1	**.033**
TMT‐B	138.87 (88.02)	24	99.09 (60.14)	22	1	**.021**
ROCF copy	30.63 (8.35)	24	32.23 (4.78)	22	1	.221
ROCF recall	13.76 (7.77)	24	16.00 (7.66)	22	0	.131

*Abbreviations*: n1, number at time point 1; n2, number at time point 2.

The multiple linear regression analysis showed that the dependent variable HVLT‐D in time point 2 was significantly influenced by age at trauma (*p*‐value = .03) and IQ (*p*‐value = .05). In this same time point, the dependent variable HVLT‐R was significantly influenced by the TB MTR (*p*‐value = .02), GM MTR (*p*‐value = .03), and WM MTR declines (*p*‐value = .04). The dependent variables TMT‐B and ROCF recall in time point 3 were significantly influenced by age at trauma (*p*‐value = .01 and *p*‐value = .02).

When the dependent variable was the variation in the test score between time point 2 and 3, HVLT‐I and HVLT‐D were significantly influenced by the TB MTR decline (*p*‐value = .01 and *p*‐value = .03), GM MTR reduction (*p*‐value = .02 and *p*‐value = .05), WM MTR decline (*p*‐value = .02 and *p*‐value = .04). HVLT‐R was also significantly influenced by the TB MTR (*p*‐value = .01), GM MTR (*p*‐value = .01), and WM MTR reductions (*p*‐value = .03). Variation in the ROCF copy test was significantly influenced by TB MTR (*p*‐value = .01), GM MTR (*p*‐value = .01), and WM MTR decrease (*p*‐value = .04). Finally, the variation in the ROCF recall test was significantly influenced by WM MTR decline (*p*‐value = .04). In our dataset, the one‐year atrophy rate did not significantly influence any of the dependent variables.

### 3.4 Correlation of neuropsychological results and MTR or volume reduction

Table 4 summarizes the Pearson or Spearman correlation regarding the neuropsychological results. The results from neuropsychological assessment in time points 2 and 3 were independently correlated with the annualized atrophy and the MTR reduction rates.

**TABLE 4:** Correlation of the brain atrophy and MTR reduction (between time points 1 and 3) and the neuropsychological tests results

**Test**	**Time point**	**TBV reduction *R* (*p*‐value)**	**GMV reduction *R* (*p*‐value)**	**WMV reduction (*p*‐value)**	**TB MTR reduction (*p*‐value)**	**GM MTR reduction (*p*‐value)**	**WM MTR reduction (*p*‐value)**
IQ	2	0.10 (.64)	0.16 (.44)	–0.09 (.64)	–‐0.12 (.56)	–‐0.21 (.30)	0.0 (.99)
HVLT‐I	2	–0.05 (.79)	0.07 (.72)	–0.13 (.53)	**–0.47 (.01)**	**–0.46 (.02)**	**–0.40 (.04)**
HVLT‐D	2	0.16 (.44)	0.35 (.09)	–0.09 (.67)	**–0.49 (.01)**	**–0.43 (.03)**	**–0.47 (.01)**
HVLT‐R	2	0.16 (.45)	0.32 (.12)	0.08 (.68)	–0.14 (.50)	–0.12 (.54)	–0.20 (.34)
TMT‐A	2	0.22 (.29)	–0.10 (.63)	**0.39 (.05)**	**0.41 (.04)**	0.34 (.09)	0.30 (.14)
TMT‐B	2	–0.03 (.88)	–0.08 (.69)	0.23 (.28)	0.25 (.24)	0.23 (.29)	0.13 (.54)
ROCF copy	2	–0.01 (.93)	–0.04 (.84)	0.20 (.35)	–0.10 (.63)	–0.18 (.38)	–0.01 (.95)
ROCF recall	2	0.05 (.81)	0.01 (.95)	0.19 (.36)	–0.23 (.27)	–0.22 (.30)	–0.13 (.53)
IQ	3	0.18 (.41)	0.07 (.75)	0.17 (.43)	0.23 (.29)	0.16 (.45)	0.30 (.16)
HVLT‐I	3	0.0 (.98)	–0.13 (.54)	0.20 (.35)	–0.02 (.92)	–0.06 (.78)	0.01 (.93)
HVLT‐D	3	0.09 (.65)	–0.10 (.68)	0.29 (.18)	0.06 (.76)	0.02 (.92)	0.09 (.68)
HVLT‐R	3	0.0 (.98)	–0.13 (.54)	0.17 (.44)	–0.00 (.99)	0.01 (.94)	–0.07 (.74)
TMT‐A	3	**0.43 (.04)**	0.22 (.31)	0.30 (.16)	0.07 (.73)	0.07 (.74)	–0.01 (.93)
TMT‐B	3	0.40 (.07)	0.06 (.78)	**0.44 (.04)**	–0.19 (.38)	–0.18 (.41)	–0.26 (.24)
ROCF copy	3	–0.03 (.87)	0.06 (.79)	–0.24 (.27)	–0.27 (.22)	–0.28 (.21)	–0.22 (.33)
ROCF recall	3	0.10 (.63)	0.08 (.69)	0.06 (.76)	–0.40 (.06)	–0.35 (.10)	**–0.44 (.03)**

Our data show a significant negative correlation of TB MTR, GM MTR, and WM MTR reductions with the HVLT‐I and HVLT‐D scores in time point 2. There was also a significant positive correlation between TB MTR decline and the TMT‐A score in time point 2 (*R* = 0.41, *p*‐value = .04). In addition, there was a significant negative correlation between WM MTR decline and ROCF recall on time point 3 (*R* = −0.44, *p*‐value = .03).

The neuropsychological results and volume data correlation evidenced a significant positive correlation between WMV atrophy and TMT‐A results in time point 2 (*R* = 0.39, *p*‐value = .05). In addition, there was a significant positive correlation between TBV atrophy and the TMT‐A score in time point 3 (*R* = 0.43, *p*‐value = .04) and between WMV atrophy and the TMT‐B score in the same time point (*R* = 0.44, *p*‐value = .04). There were no other significant correlations between the volume or the MTR reduction and the neuropsychological results.

### 3.5 Correlation of MTR decrease and volume atrophy

Table 5 depicts the results of the Pearson or Spearman correlation between MTR decline and volume reduction. There was no association between volume reduction and MTR decrease for the TB, GM, or WM in any of the intervals studied.

**TABLE 5:** Pearson's correlation results (Normality = 0) and Spearman's correlation results (Normality = 1) between volume reduction and MTR reduction in each encephalic compartment

**Compartment**	**Normality**	**Time points compared**	**Correlation coefficient (*R*)**	***p*‐value**
TB	0	**1** **vs. 2**	–0.01	.926
	1	**1** **vs. 3**	0.17	.410
	1	**2** **vs. 3**	0.24	.247
GM	0	**1** **vs. 2**	0.15	.451
	1	**1** **vs. 3**	0.12	.536
	0	**2** **vs. 3**	–0.02	.901
WM	0	**1** **vs. 2**	0.08	.703
	1	**1** **vs. 3**	0.11	.581
	1	**2** **vs. 3**	0.20	.325

*Abbreviations*: GM, gray matter; TB, total brain; WM, white matter.

### 3.6 Correlation of the variation between neuropsychological results and imaging results

Table 4 shows the Pearson or Spearman results when the variance in the neuropsychological scores (between time points 2 and 3) was correlated to the MTR or volume decline during the same interval. The WM MTR reduction between time points 2 and 3 was negatively correlated with the IQ increase (*R* = −0.51, *p*‐value = .01) and with the improvement in the TMT‐A scores (*R* = −0.43, *p*‐value = .04). The TBV atrophy throughout this period was significantly correlated with the variance in the TMT‐A (*R* = 0.60, *p*‐value = 0) and the ROCF copy test (*R* = 0.44, *p*‐value = .04). In addition, there was a significant negative correlation between the variance in the ROCF recall test and the GM atrophy (*R* = −0.51, *p*‐value = .01). There were no additional significant correlations between the reduction in volume or MTR and the variance in neuropsychological outcomes. Table 6

**TABLE 6:** Correlation of the brain atrophy and MTR reduction (between time points 2 and 3) and the variance between the neuropsychological tests results

**Test**	**TBV reduction *R* (*p*‐value)**	**GM reduction *R* (*p*‐value)**	**WM reduction *R* (*p*‐value)**	**TB MTR reduction *R* (*p*‐value)**	**GM MTR reduction *R* (*p*‐value)**	**WM MTR reduction *R* (*p*‐value)**
IQ	–0.19 (.38)	0.05 (.81)	–0.28 (.20)	–0.41 (.05)	–0.36 (.10)	**–0.51 (.01)**
HVLT‐I	–0.15 (.51)	0.13 (.56)	–0.41 (.06)	–0.28 (.21)	–0.24 (.28)	–0.16 (.48)
HVLT‐D	–0.25 (.27)	0.16 (.47)	–0.36 (.10)	–0.20 (.36)	–0.18 (.42)	–0.06 (.78)
HVLT‐R	0.18 (.41)	0.27 (.22)	–0.11 (.63)	–0.05 (.82)	–0.08 (.72)	0.02 (.91)
TMT‐A	**0.60 (0)**	0.24 (.28)	0.39 (.08)	0.36 (.09)	0.40 (.06)	**0.43 (.04)**
TMT‐B	0.34 (.15)	0.15 (.52)	0.19 (.41)	0.16 (.49)	0.15 (.53)	0.18 (.45)
ROCF copy	**0.44 (.04)**	0.11 (.62)	0.32 (.15)	–0.02 (.92)	–0.02 (.91)	0.08 (.72)
ROCF recall	–0.02 (.89)	**–0.51 (.01)**	0.25 (.27)	0.30 (.17)	0.22 (.32)	0.22 (.33)

*Abbreviation*: R, correlation coefficient.

## 4 DISCUSSION

This study characterizes a progressive decline in the WMV and TBV volume up to one year after TBI, with an annualized TBV atrophy rate of 1.38% and a WMV rate of 3.61%. These values are significantly higher than the annual atrophy rates of up to 0.52% for the TB and 0.47% for the WM in normal individuals (Ge et al., 2002; Smith et al., 2007). The annualized WMV atrophy rate matches the atrophy rate of around seven years of normal aging. In addition, the annualized TBV atrophy rate matches the 1.5% rate described in moderate and severe TBI, but not for the WM, in which the reported atrophy rate is around 1.4% (Ding et al., 2008; Sidaros et al., 2009; Trivedi et al., 2007).

We also describe a progressive decline of the mean TB MTR, GM MTR, and WM MTR, which reflects the Wallerian degeneration of axons, mitochondrial dysfunction, excitotoxicity, oxidative stress, and the apoptotic cell death of neurons and glia observed in the delayed phase after TBI (Ng et al., 2019). The interval of more significant WM MTR decline (between time points 1 and 2) preceded the one with greater WMV atrophy (between time points 2 and 3) and no correlation was found between MTR and volume reductions throughout the study. The relative independence of MTR decline and atrophy could be explained by pathological correlations that suggest that, despite atrophy being associated with MTR decline, demyelination is the primary driver of MTR reduction. (Foss et al., 2013). It could also be reinforced by histological research in rats with induced encephalomyelitis, which revealed no association between cortical demyelination and atrophy, implying that the correlation between myelinic loss and parenchymal loss is complex (Pomeroy et al., 2008).

Our follow‐up also evidenced a significant improvement in the HTLV‐I, TMT‐A, and TMT‐B scores between time points 2 and 3. In addition, improvement in the TMT‐A scores showed a strong correlation with the WM MTR decline and TBV atrophy. The improvement in TMT results agrees with a previous study that evidenced improvement in TMT‐A and TMT‐B after a year post‐trauma (Farbota et al., 2012). After a brain injury, partial or complete recovery of specific cognitive domains can be attributed to both the restoration and relearning of lost capabilities and adaptation and compensating of spared functions (Nudo, 2013).

Previous research has revealed that patients with TBI often have an amnesic pattern characterized by verbal episodic memory deficits, particularly in late recollection of new material (Vakil, 2005). However, there is disagreement regarding the period of most significant cognitive recovery after a TBI; some believe it to be 6–12 months, while others suggest the interval between 1 month to 1 year after the trauma (Dikmen et al., 1987). Patients with severe TBI exhibited improved verbal episodic memory scores when tested six and twelve months after the event (Kersel et al., 2001). Nonetheless, even after recovery, patients are unlikely to regain their premorbid ability levels and continue to score worse than healthy people compared to normative data (Zaninotto et al., 2017).

In general, in this study, neuropsychological scores correlated more with MTR decline than with volume atrophy. In healthy individuals, studies have described a significant correlation between TB MTR and cortical GM MTR and performance on memory, executive function, and motor skills assessments (Seiler et al., 2014). Lower MTR in the normal‐appearing white matter has also been correlated with impairments in processing speed, executive function, and episodic memory in normal adults aged 50−90 years (Schiavone et al., 2009). In mild TBI, a significant correlation between the California Verbal Learning Test results and the MTR values for the splenium and between the Wechsler Memory Scale‐Visual Span Forward subtest and the MTR value for the pons was described (McGowan et al., 2000). However, partial volume effects related to the ROI analysis might have underestimated the results in this latter. Up to our knowledge, there is no study comparing MTR values and neuropsychological results in patients with moderate and severe DAI.

The main limitations of this work include the relatively small sample size and the gender imbalance. The sample size was probably one of the main contributors for not finding a significant relationship between the time after trauma and MTR or volume decline, despite significant differences in the MTR and volume mean between time points 1 and 3. The gender imbalance is common in TBI studies since males are more frequently affected by TBI than females. However, studies investigating brain atrophy in TBI patients that used controls matched by gender found similar results to others that used unmatched controls (Ding et al., 2008; Farkas et al., 2007; Kim et al., 2008; Trivedi et al., 2007). The inclusion of both moderate and severe TBI. Another limitation is inherent to MTI. Even though MTR is considered sensitive to alterations in the microstructural environment, its ability to reveal specific forms of underlying pathology requires further investigation, particularly in TBI, where multiple forms of pathology may co‐exist (Pagani et al., 2008).

To our knowledge, this is the first longitudinal study to assess patients with DAI using MTR and volumetry. It is also the first one to obtain global MTR instead of in small ROI and to compare MTR and neuropsychological results in DAI. In addition, this work introduces a simple pre‐processing methodology for automatic extraction of hemorrhagic content. Blood content is a significant inconvenience for many quantitative advanced MRI techniques, such as MTI and Diffuse Tensor Imaging (DTI). Therefore, excluding blood content should be a fundamental step in the processing pipeline to obtain reliable and reproducible results.

## 5 CONCLUSION

On a series of MRIs performed up to one year after DAI, this study shows gradual axonal/myelinic rarefaction and volume loss. Despite a tendency toward worsening MRI quantitative measures, neuropsychological tests revealed that some patients had improved episodic verbal memory, attention, and executive function, suggesting that at least part of the morphometric changes result from a dynamic process in which reorganization prevails over the damage.

## CONFLICT OF INTEREST

The authors declare no conflict of interest.

### PEER REVIEW

The peer review history for this article is available at https://publons.com/publon/10.1002/brb3.2490

## Data Availability

Research data are not available for sharing, since it contains patient information.
